# Skin accumulation of advanced glycation end-products predicts kidney outcomes in type 2 diabetes: results from the Brazilian Diabetes Study

**DOI:** 10.1590/2175-8239-JBN-2024-0047en

**Published:** 2024-08-23

**Authors:** Joaquim Barreto, Marilia Martins, Cynthia M. Borges, Sofia Helena Vitte, Wilson Nadruz, Rodrigo Bueno de Oliveira, Andrei C. Sposito

**Affiliations:** 1 Universidade Estadual de Campinas, Área de Cardiologia, Laboratório de Aterosclerose e Biologia Vascular, São Paulo, SP, Brazil.; 2 Universidade Estadual de Campinas, Área de Nefrologia, Laboratório para Avaliação do Distúrbio Mineral e Ósseo em Nefrologia, São Paulo, SP, Brazil.; 3 Universidade Estadual de Campinas, Área de Cardiologia, São Paulo, SP, Brazil.

**Keywords:** Renal Insufficiency, Chronic, Glycation End Products, Advanced, Diabetes Mellitus, Type 2

## Abstract

The accumulation of advanced glycation end-products (AGEs) elicits morphofunctional kidney impairment. AGEs levels can be noninvasively estimated by skin autofluorescence (SAF). We explored whether high SAF predicts kidney outcomes in type 2 diabetes (T2D) individuals. The study was conducted as a predefined analysis of the Brazilian Diabetes Study, a prospective single-center cohort of T2D adults. Data from 155 individuals followed for up to 1716 days were considered. The incidence of major adverse kidney events (MAKE) was 9.6%. Individuals with above-median SAF had a higher incidence of MAKEs (4.6% *vs.* 21%; p = 0.002), with an HR of 3.39 [95% CI: 1.06–10.85; p = 0.040] after adjustment by age and gender. The mean adjusted eGFR change was 1.08 units (SE: 1.15; 95%CI: –1.20, 3.37) in the low SAF and –5.19 units [SE: 1.93; 95%CI: –9.10, –1.29] in the high SAF groups (between-subject difference: F: 5.62, p = 0.019). The high-SAF group had a greater prevalence of rapid decliners than the low-SAF group (36.7% *vs.* 15.8%; p = 0.028). In conclusion, high SAF was related to increased incidence of MAKEs and faster decline in eGFR among T2D subjects. This should be considered by healthcare providers when identifying individuals more prone to diabetes-related kidney complications.

## Introduction

Chronic kidney disease (CKD) affects one-third of individuals with diabetes, and it increases the risk of mortality and disability^1^. As diabetes prevalence rises globally, now exceeding 573 million cases, the identification of individuals predisposed to kidney complications is paramount to reduce mortality^2^.

The assessment of advanced glycation end-products (AGEs) accumulated in the skin, measured conveniently through skin autofluorescence (SAF), emerges as a promising strategy to identify diabetic and non-diabetic individuals at risk of AGE-related complications^3,4^. In fact, previous experimental data indicate AGE accumulation in the kidney, triggering podocyte apoptosis, mesangial morphofunctional impairment, and vascular hyperpermeability, all of which contribute to accelerated kidney function decline^5^.

In the context of CKD, AGE accumulation can be exacerbated by their increased formation due to oxidative and carbonyl stress with concomitant deterioration of kidney function. These chemically stable AGEs bound to long-lived proteins reflect cumulative metabolic stress, that perpetuate cellular damage by direct protein modification, functional alterations, or binding to specific AGEs receptors (RAGEs), inducing a range of cellular responses that culminate in injury^6^. Consequently, AGEs represent potential biomarkers for the evaluation of kidney disease progression in CKD^7,8^ (Figure S1).

Despite this, the predictive value of increased SAF for adverse kidney outcomes is underexplored, particularly in the Brazilian population, which has unique demographic, ethnic, and epidemiological characteristics. To address this gap, we investigated the association between SAF levels and the incidence of kidney outcomes among individuals with T2D in a tertiary research center.

## Methods

This study was a predefined analysis of the Brazilian Diabetes Study (clinicaltrials.gov: NCT04949152), a prospective cohort of T2D held by a medical research center in Brazil. The cohort profile has been published previously and is detailed in the supplementary material^9^. SAF was assessed at baseline, whereas blood and urinary samples were collected at baseline and regularly thereafter to assess outcomes.

The AGE-ReaderTM (DiagnOptics BV, Groningen, The Netherlands) device was used to estimate the concentration of AGEs in the skin based on the emitted fluorescence, which was calculated in triplicate on the ventral side of the forearm^10^. Only individuals with a 6% photo-type skin reflection index (Fitzpatrick class I to IV) were considered. As no validated SAF reference value exists for our population, participants were grouped as high or low SAF based on the value with the highest accuracy for the outcome of interest in receiver operation curve (ROC) analysis^10^.

Total blood samples were collected in 4-mL EDTA vacuum tubes and centrifuged at 3500 rev/min for 10 min at 4°C. Plasma was aliquoted in cryotubes and frozen at –80°C until analysis. The estimated glomerular filtration rate (eGFR) was calculated using the serum creatinine levels and the CKD Epidemiology Collaboration equation (CKD-EPI), as previously validated^11^. Spot urinary samples were analyzed for the measurement of creatinine and albumin levels. Albumin-to-creatinine ratio above 30 mg/g was considered abnormal^12^.

The primary outcome was the difference between SAF groups in the incidence of major adverse kidney events (MAKEs), defined as new-onset of any of the following: persistent proteinuria, eGFR <60 mL/min/1.73 m^2^, eGFR decrease of more than 40% from baseline, end-stage kidney disease as eGFR<15 mL/min/1.73 m^2^ or renal replacement therapy initiation, and death from a renal cause^13^. Secondary outcomes were: (i) the difference between groups in the mean annualized change in eGFR from baseline to the last eGFR measurement; (ii) the difference in the prevalence of rapid decliners, defined as individuals with an annualized eGFR drop greater than 5 mL/min/1.73 m^2 12,14^.

### Statistical Analysis

Means were compared by Student’s t-test when data were normally distributed and by the Mann-Whitney test when data were non-normally distributed. According to the SAF value, the hazard ratio (HR) of MAKE was estimated by Cox regression and adjusted by covariates. Repeated measures ANOVA was used to compare the between-subjects eGFR variation across SAF groups. The annualized eGFR change between the first and last eGFR measures was ranked to suit normality and compared by RANCOVA using SAF as the independent variable. ROC and the concordance (C) statistic were used to analyze the ability of SAF to predict MAKE. All analyses were performed using the SPSS 28.0 version for MAC, and the p-value <0.05 was considered significant.

## Results

Data from 155 individuals that were followed for up to 1716 days (median: 971; IQR: 550; range: 28–1716 days) were considered. This population had a mean age of 58 ± 7 years, 64% were male, and median T2D duration was 9 years (IQR: 10.6). The prevalence of prior cardiovascular disease (CVD) was 19.4% (18.1% coronary heart disease and 1.3% prior stroke), 3.2% were active smokers, and 91% had hypertension. Mean systolic and diastolic blood pressure was 144 ± 21 mmHg and 85 ± 12 mmHg, respectively, body mass index (BMI) was 30 ± 4.6 kg/m^2^, and mean glycated hemoglobin (HbA1c) was 8 ± 1.6 %. The baseline eGFR was 86 mL/min/1.73 m^3^ (grade 1: 50%; grade 2: 42.6%; grade 3A: 4.7%; grade 3B: 1.4%; grade 4: 1.4%), and 14.8% had abnormal albuminuria (A1: 34.8%; A2: 11.6%; A3: 3.2%).

During the follow-up time, 15 (9.7%) MAKEs were reported: 10 incident CKD (6.5%), 1 (0.1%) eGFR decline >40%, and 4 new-onset proteinuria (2.6%).

The MAKE group had higher values of SAF [2.50 (IQR: 0.7) *vs*. 3.02 (IQR: 0.8); p = 0.008] and BMI [29 ± 5.6 *vs.* 32 ± 6.8; p = 0.008] and had a higher prevalence of prior CVD (16.4% *vs*. 40%; p = 0.033) compared to with no MAKE (Table 1). In univariate Cox regression, SAF [HR: 2.847; 95% CI: 1.308, 6.197; p = 0.008, per unit], CVD [HR: 3.23; 95% CI: 1.14–9.10; p = 0.027], and BMI [HR: 1.142; 95% CI: 1.035–1.260; p = 0.008] were correlated to MAKE. Furthermore, SAF [HR: 3.937; 95% CI: 1.707–9.081; p = 0.001] and BMI [HR: 1.176; 95% CI; 1.064–1.300; p = 0.001], but not CVD (HR: 2.849; 95% CI: 0.932–8.706; p = 0.066), were independently correlated to MAKE after adjustment by covariates.

**Table 1:** Baseline characteristics according to kidney outcome status

	No adverse kidney outcomes (n = 140)	Adverse kidney outcomes (n = 15)	p-value
Age, years	59 (11)	63 (10)	0.126
Male, %	86 (61)	13 (87)	0.053
T2D duration, years	8.5 (11)	7.6 (11)	0.514
Hypertension, %	126 (90)	15 (100)	0.199
Dyslipidemia, %	123 (88)	15 (100)	0.153
CVD, %	24 (17)	6 (40)	**0.033**
Smoking, %	5 (3.6)	0 (0)	0.457
Former smoker, %	57 (41)	11 (73)	**0.016**
SBP, mmHg	142 (26)	140 (22)	0.439
DBP, mmHg	84 (17)	86 (10)	0.445
BMI	29 (5.6)	32 (6.8)	**0.008**
SAF, AU	2.5 (0.7)	3 (0.8)	**0.008**
Biochemical analysis			
Hb, g/dL	14 (1.7)	15 (2.5)	0.162
A1c, %	7.6 (1.6)	7.7 (1.7)	0.730
Total cholesterol, mg/dL	172 (55)	140 (58)	0.120
LDL - C, mg/dL	100 (41)	75 (50)	0.074
HDL - C, mg/dL	43 (14)	34 (19)	0.852
VLDL - C, mg/dL	27 (12)	32 (11)	0.466
Triglycerides, mg/dL	163 (88)	218 (174)	0.159
Creatinine, mg/dL	0.9 (0.3)	0.9 (0.4)	0.074
eGFR, mL/min	87 (23)	87 (34)	0.187
GFR Class, %			0.259
G1	68 (51)	6 (40)	
G2	55 (41)	8 (53)	
G3a	7 (5.3)	0 (0)	
G3b	1 (0.8)	1 (7)	
G4	2 (1.5)	0 (0)	
G5	0	0	
eGFR < 60 mL/min, %	10 (7.5)	1 (7)	0.905
Proteinuria, %	32 (24)	5 (36)	0.348
Albuminuria class, %			0.201
A1	52 (37)	2 (13.3)	
A2	15 (11)	3 (20)	
A3	5 (3.6)	0 (0)	

Abbreviations: T2D: type 2 diabetes; CVD: cardiovascular disease; SBP: Systolic blood pressure; DBP: diastolic blood pressure; BMI: body mass index; SAF: skin autofluorescence; AU: arbitrary units; LDL - C: low-density lipoprotein; HDL - C: high-density lipoprotein; VLDL - C: very low-density lipoprotein; eGFR: estimated glomerular filtration rate. Notes: eGFR and albuminuria class according to the KDIGO classification^7^. CVD for prior coronary heart disease (prior acute coronary syndrome, stable angina, or myocardial revascularization) or stroke.

SAF had an area under curve (AUC) of 0.786 (SE: 0.067; 95% CI: 0.654–0.917; p < 0.001) for MAKE. K-S statistics returned the optimal SAF cut-off value of 2.85 AU (sensitivity: 80%, specificity: 26%) and a max non-parametric Kolmogorov-Smirnoff test of 0.538, indicating that SAF was better in predicting renal outcomes for this population than a random model.

**Figure 1:**
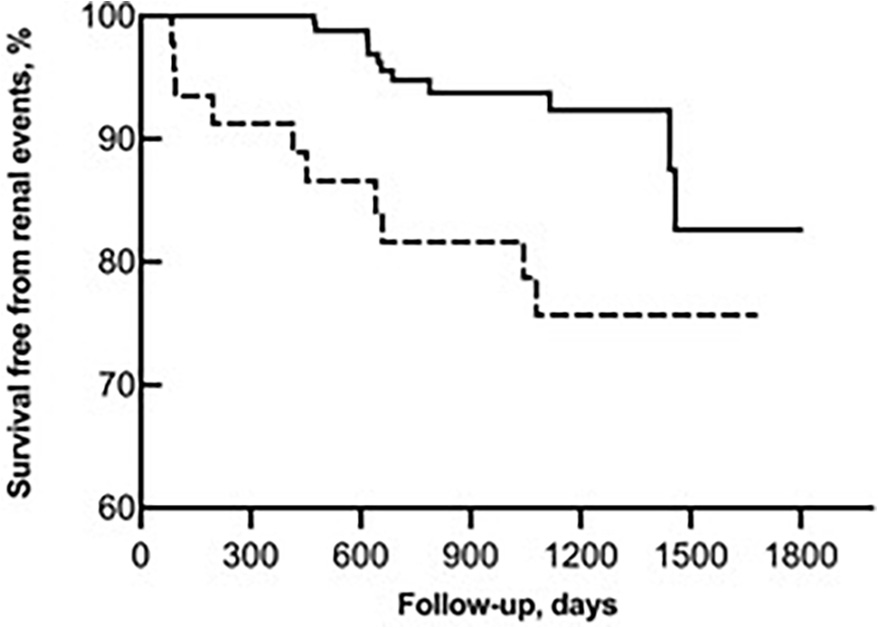
Incident MAKE according to SAF group.

Based on this finding, we grouped patients as high SAF (n = 46; ≥ 2.85 AU) and low SAF (n = 109; <2.85 AU). Compared to the low SAF, the high SAF group were older (57 ± 7 years *vs*. 61 ± 4.9 years; p < 0.001) and had higher frequency of males [57.8% *vs*. 78.3%; p = 0.015] (see supplementary Table S1). The low SAF group had 5 (4.6%) and high SAF had 10 (21.7%) MAKEs (p = 0.002), with an HR of 4.58 (95% CI: 1.56–13.46; p = 0.006) in the unadjusted model and an HR of 3.39 [95% CI: 1.06–10.85; p = 0.040] after adjustment by age and gender (Figure 1). Further adjustment by systolic blood pressure and HbA1c, which are known risk factors for MAKEs, did not abrogate the influence of high SAF on outcomes [HR: 5.28; 95% CI: 1.44, 19.37; p = 0.012].

**Figure 2:**
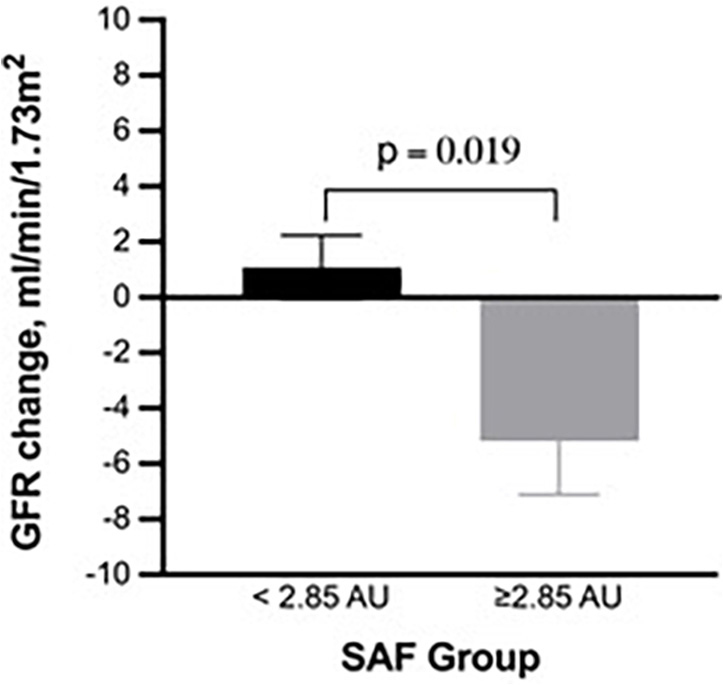
Mean adjusted change in glomerular filtration rate according to SAF groups.

At baseline, eGFR was comparable between SAF groups. eGFR levels were unchanged in the low SAF group (87 + 18 units *vs*. 88 + 18 units; p = 0.078), whereas eGFR significantly decreased among the high SAF individuals (83 + 17 units vs 78 + 19 units; p = 0.025). The mean adjusted eGFR change was 1.08 units (SE: 1.15; 95% CI: –1.20, 3.37) in the low SAF and –5.19 units [SE: 1.93; 95%CI: –9.10, –1.29] in the high SAF groups (between-subjects difference: F: 5.62, p = 0.019) (Figure 2). The high SAF group had a greater prevalence of rapid decliners than the low SAF group (36.7% *vs.* 15.8%; p = 0.028).

## Discussion

The accumulation of AGEs in the skin provides a feasible and reproducible method for identifying individuals at higher risk for adverse kidney outcomes. Our study demonstrated that high SAF values were an independent risk factor for MAKEs and predicted a faster eGFR decline. These findings align with prior reports from retrospective studies conducted in Eastern countries, which showed that eGFR decline rate and incident of end-stage kidney disease are associated with higher SAF values in both non-T2D and T2D subjects with established CVD^8,15^. As the progression of kidney impairment varies significantly across countries and is influenced by the prevalence of CVD, our data add to prior results as it validates this relationship in an ethnically diverse population with a broader cardiovascular risk spectrum^16^. SAF values can vary significantly across populations, and this underscores the need for region-specific reference values, as demonstrated by previous research^17^.

In earlier studies, the predictive value of SAF was described for diabetes complications. Boersma et al.^18^ confirmed that SAF predicts CVD and mortality in a larger group of 2349 people with T2D. SAF also showed a stronger association with future cardiovascular events (CVEs) and mortality than cholesterol or blood pressure levels. Kidney failure is another common condition associated with AGEs accumulation and is clinically associated with a strongly increased rate of cardiovascular complications. SAF was also identified as an independent and robust predictor of mortality. Compared with variables in the UK Prospective Diabetes Study (UKPDS) risk engine, SAF was considered as the most effective single predictor of total and cardiovascular mortality after age, adding predictive value to the UKPDS risk engine, resulting in risk reclassification in 25–30% of patients^19^.

Initial studies on SAF in kidney failure reported increased SAF levels in patients with moderate kidney failure up to hemodialysis patients and confirmed SAF as a strong and independent risk predictor for mortality in patients with renal failure^20,21^ and in transplant patients^22^. Arsov et al.^23^ in 2014 and Crowley et al.^24^ in 2013 found strongly increased SAF levels in HD patients. In the latter study, SAF levels were 64% higher than in age-matched controls, with a decrease in SAF levels after kidney transplantation. In summary, T2D individuals with higher SAF values are at a greater risk of MAKE. Further studies are needed to determine whether treatment to reduce AGE accumulation results in improved kidney outcomes.

Our study had limitations. Firstly, the sample size was small compared to previous studies, resulting in a limited number of events, which may have affected statistical power. Secondly, the cut-off value used for SAF may not be applicable to other populations, as this variable is susceptible to the influence of demographic characteristics that vary significantly across studies. Finally, while the annualized eGFR decline rate is more accurate when eGFR values are measured at regular intervals, we calculated the eGFR slope based on serum creatinine measures opportunistically assessed during the study.

## Supplementary Material

The following online material is available for this article:

Supplementary Methods – Brazilian Diabetes Study.
