# Supplementary material for: Skin accumulation of advanced glycation end-products predicts kidney outcomes in type 2 diabetes: results from the Brazilian Diabetes Study
Source: J Bras Nefrol. 2024 Aug 23;46(4):e20240047. doi: 10.1590/2175-8239-JBN-2024-0047en (PMC11346773; doi:10.1590/2175-8239-JBN-2024-0047en)
Supplement: Supplementary file 1 [file 2175-8239-jbn-46-4-e20240047-s1.pdf]

*Braz. J. Nephrol. (J. Bras. Nefrol.)*
DOI: https://doi.org/10.1590/2175-8239-JBN-2024-0047en

# Supplementary Material to "Skin accumulation of advanced glycation end-products predicts kidney outcomes in type 2 diabetes: results from the Brazilian Diabetes Study"

## Supplementary Methods - Brazilian Diabetes Study

The Brazilian Diabetes Study is an ongoing, prospective cohort of individuals with type 2 diabetes (T2D) aged 30 years or older, held by the Laboratory of Atherosclerosis and Vascular Biology of the University of Campinas (Unicamp), Brazil. This study was approved by the local ethics committee (CAEE: 89525518.8.1001.5404) and is registered in the clinicaltrials.gov database (NCT04949152). The study design is described in detail elsewhere[1]. In brief, participants were invited to attend the clinical research center for study design clarification and presentation of the informed consent form. On this occasion, participants were interviewed by a licensed physician-researcher about their medical history, current medications in use, and baseline anthropometric and demographic data.

Participants underwent skin autofluorescence (SAF) measurement using the AGE-Reader™ (DiagnOptics BV, Groningen, The Netherlands), which estimates the concentration of advanced glycation end-products (AGE) from the fluorescence emitted by the skin, calculated in triplicate on the ventral side of the forearm. Only subjects with a 6-percent photo-type skin reflectance index (Fitzpatrick class I to IV) were considered for this analysis. SAF values are presented as arbitrary units (AU). As there is no validated SAF reference value for our population, participants were categorized into high or low SAF groups based on the SAF value with the highest accuracy for the outcome of interest in the receiver operating characteristic curve (ROC) analysis[2].

At baseline, participants underwent blood and urine sampling and biochemical analysis to assess complete blood count, glycemic control, lipid profile, serum creatinine, and urinalysis including determination of urinary albumin levels. Glomerular filtration rate (GFR) was estimated based on the previously validated CKD-EPI equation[3]. Proteinuria was

considered in all individuals who had a urinary protein-to-creatinine ratio greater than 0.2 mg/g or an albumin-to-creatinine ratio greater than 30 mg/g in urine spot[4]. The eGFR groups were defined according to the KDIGO classification system as follows: G1 (≥90 mL/min/m$^2$), G2 (60-90 mL/min/m$^2$), G3a (45-60 mL/min/m$^2$), G3b (30-45 mL/min/m$^2$), G4 (15-30 mL/min/m$^2$), and G5 (<15 mL/min/m$^2$). All biochemical analyses were performed at baseline and repeated as appropriate during the follow-up period.

The primary outcome was the difference between SAF groups in the incidence of the composite of major adverse kidney events (MAKEs), defined as new-onset of any of the following events: persistent proteinuria, eGFR < 60 mL/min/m$^2$, eGFR decline of more than 40% from baseline, end-stage kidney disease as eGFR < 15 mL/min/m$^2$ or initiation of renal replacement therapy, and death from kidney cause[5]. Secondary outcomes were: (i) the difference between groups in the mean annualized change in eGFR from baseline to the last eGFR measurement; (ii) the difference in the prevalence of rapid decliners, defined as individuals with an annualized eGFR decline of more than 5 mL/min/m$^2$.[4,6]

**Table S1.** Baseline characteristics according to SAF group.

| | **SAF** | | |
|---|---|---|---|
| | **< 2.85 AU** | **≥ 2.85 AU** | **p-value** |
| n | 106 | 49 | |
| Age, years | 57 ± 7.3 | 61 ± 4.9 | < 0.001 |
| Male, % | 63 (57.8) | 36 (78.3) | 0.015 |
| T2D duration, years | 8.54 (10.2) | 8.00 (15.1) | 0.842 |
| Hypertension, % | 98 (89.9) | 43 (93.5) | 0.479 |
| Dyslipidemia, % | 98 (89.9) | 40 (87) | 0.591 |
| CVD, % | 17 (15.6) | 13 (28.3) | 0.068 |
| Smoking, % | 4 (3.7) | 1 (2.2) | 0.630 |

| | SAF | | |
|---|---|---|---|
| | < 2.85 AU | ≥ 2.85 AU | p-value |
| Former smoker, % | 44 (40.4) | 24 (52.2) | 0.176 |
| SBP, mmHg | 143 ± 20.9 | 151 ± 21.1 | 0.050 |
| DBP, mmHg | 85.6 ± 13.3 | 84.1 ± 10.1 | 0.631 |
| BMI | 30.4 ± 4.61 | 30.6 ± 4.51 | 0.805 |
| SAF, AU | 2.30 (0.50) | 3.25 (0.60) | 0.002 |
| ***Biochemical analysis*** | | | |
| Hb, g/dL | 14.3 ± 1.59 | 14.0 ± 1.39 | 0.425 |
| A1c, % | 8.12 ± 1.64 | 7.78 ± 1.51 | 0.227 |
| Total cholesterol, mg/dL | 178 ± 42.4 | 178 ± 46.9 | 0.968 |
| LDL-C, mg/dL | 104 ± 35.9 | 105 ± 37.9 | 0.879 |
| HDL-C, mg/dL | 43.1 ± 10.6 | 43.2 ± 12.4 | 0.956 |
| VLDL-C, mg/dL | 27 (14.8) | 28 (12) | 0.628 |
| Triglycerides, mg/dL | 169 (131) | 171 (88) | 0.828 |
| Creatinine, mg/dL | 0.90 ± 0.30 | 0.95 ± 0.21 | 0.364 |
| eGFR, mL/min | | | |
| Baseline | 87 ± 18.6 | 83.3 ± 17.3 | 0.216 |
| Final | 88.1 ± 18.4 | 78.1 ± 19.9 | 0.004 |
| Change | 1.08 (1.15) | -5.19 (1.93) | 0.019 |
| eGFR Class, % | | | 0.616 |

| | SAF | | |
|---|---|---|---|
| | < 2.85 AU | ≥ 2.85 AU | p-value |
| G1 | 53 (51.5) | 21 (46.7) | |
| G2 | 42 (40.8) | 21 (46.7) | |
| G3a | 4 (3.9) | 3 (6.7) | |
| G3b | 2 (1.9) | 0 | |
| G4 | 2 (1.9) | 0 | |
| G5 | 0 | 0 | |
| eGFR < 60 mL/min, % | 8 (7.8) | 3 (6.7) | 0.814 |
| Proteinuria, % | 21 (20.6) | 16 (36.4) | 0.071 |
| Albuminuria class, % | | | 0.163 |
| A1 | 44 (40.4) | 10 (21.7) | |
| A2 | 11 (10.1) | 7 (15.2) | |
| A3 | 3 (2.8) | 2 (4.3) | |

T2D: type 2 diabetes; CVD: cardiovascular disease; SBP: Systolic blood pressure; DBP: diastolic blood pressure; BMI: body mass index; SAF: skin autofluorescence; AU: arbitrary units; LDL-C: low-density lipoprotein; HDL-C: high-density lipoprotein; VLDL-C: very low-density lipoprotein; eGFR: estimated glomerular filtration rate. eGFR and albuminuria class according to the KDIGO classification CVD for prior coronary heart disease (prior acute coronary syndrome, stable angina, or myocardial revascularization) or stroke.

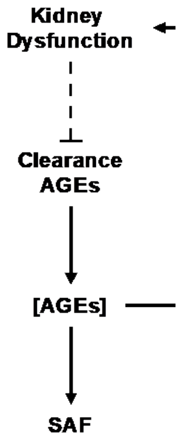


**Figure S1.** Kidney dysfunction and SAF.

Kidney dysfunction attenuates the clearance of AGEs, which perpetuate kidney outcomes and accumuilate in the skin, being is assessed by SAF.

**REFERENCES**


1. Barreto J, Wolf V, Bonilha I, Luchiari B, Lima M, Oliveira A, et al. Rationale and design of the Brazilian diabetes study: a prospective cohort of type 2 diabetes. Curr Med Res Opin. 2022;38(4):523-9. doi: http://doi.org/10.1080/03007995.2022.2043658. PubMed PMID: 35174749.

2. Fonseca LF, Araujo AB, Quadros K, Carbonara CEM, Dertkigil SSJ, Sposito AC, et al. AGEs accumulation is related to muscle degeneration and vascular calcification in peritoneal dialysis patients. J Bras Nefrol. 2021;43(2):191-9. doi: http://doi.org/10.1590/2175-8239-jbn-2020-0119. PubMed PMID: 33650629.

3. Levey AS, Stevens LA, Schmid CH, Zhang YL, Castro AF 3rd, Feldman HI, et al. A new equation to estimate glomerular filtration rate. Ann Intern Med. 2009;150(9):604-12. doi: http://doi.org/10.7326/0003-4819-150-9-200905050-00006. PubMed PMID: 19414839.

4. Matsushita K, van der Velde M, Astor BC, Woodward M, Levey AS, de Jong PE, et al. Association of estimated glomerular filtration rate and albuminuria with all-cause and cardiovascular mortality in general population cohorts: a collaborative meta-analysis. Lancet. 2010;375(9731):2073-81. doi: http://doi.org/10.1016/S0140-6736(10)60674-5. PubMed PMID: 20483451.

5. Prischl FC, Rossing P, Bakris G, Mayer G, Wanner C. Major adverse renal events (MARE): a proposal to unify renal endpoints. Nephrol Dial Transplant. 2021;36(3):491-7. doi: http://doi.org/10.1093/ndt/gfz212. PubMed PMID: 31711188.

6. Oshima M, Shimizu M, Yamanouchi M, Toyama T, Hara A, Furuichi K, et al. Trajectories of kidney function in diabetes: a clinicopathological update. Nat Rev Nephrol. 2021;17(11):740-50. doi: http://doi.org/10.1038/s41581-021-00462-y. PubMed PMID: 34363037.
