# Supplementary material for: Skin accumulation of advanced glycation end-products predicts kidney outcomes in type 2 diabetes: results from the Brazilian Diabetes Study
Source: J Bras Nefrol. 2024 Aug 23;46(4):e20240047. doi: 10.1590/2175-8239-JBN-2024-0047en (PMC11346773; doi:10.1590/2175-8239-JBN-2024-0047en)
Supplement: Supplementary file 2 [file 2175-8239-jbn-46-4-e20240047-s2.pdf]

Braz. J. Nephrol. (J. Bras. Nefrol.)
DOI: https://doi.org/10.1590/2175-8239-JBN-2024-0047pt

Material Suplementar para "Acúmulo de produtos finais de glicação avançada na pele prediz desfechos renais em diabetes tipo 2: resultados do Estudo Brasileiro sobre Diabetes"

Métodos Suplementares - Estudo Brasileiro sobre Diabetes

O Estudo Brasileiro sobre Diabetes é uma coorte prospectiva, em andamento, de indivíduos com diabetes tipo 2 (DT2) com 30 anos ou mais, realizada pelo Laboratório de Aterosclerose e Biologia Vascular da Universidade de Campinas (Unicamp), Brasil. Este estudo foi aprovado pelo comitê de ética local (CAEE: 89525518.8.1001.5404) e está registrado no banco de dados clinicaltrials.gov (NCT04949152). O desenho do estudo está descrito de forma detalhada em outro material[1]. Em resumo, os participantes foram convidados a comparecer ao centro de pesquisa clínica para esclarecimentos sobre o desenho do estudo e apresentação do termo de consentimento livre e esclarecido. Na ocasião, os participantes foram entrevistados por um médico pesquisador licenciado sobre seu histórico médico, medicamentos atualmente em uso e dados antropométricos e demográficos basais.

Os participantes foram submetidos à medição da autofluorescência da pele (sAF) utilizando o AGE-Reader™ (*DiagnOptics BV*, Groningen, Holanda), que estima a concentração de produtos finais de glicação avançada (AGEs) a partir da fluorescência emitida pela pele, calculada em triplicata na face ventral do antebraço. Somente indivíduos com um índice de refletância da pele com fototipo de 6% (classe I a IV de Fitzpatrick) foram considerados para essa análise. Os valores de sAF são apresentados como unidades arbitrárias (UA). Como não existe um valor de referência de sAF validado para nossa população, os participantes foram categorizados em grupos de sAF elevada ou baixa com base no valor de sAF com a maior precisão para o desfecho de interesse na análise da curva ROC (*Receiver Operating Characteristic*)[2].

No início do estudo, os participantes foram submetidos a coleta de sangue e urina e análise bioquímica para avaliar hemograma completo, controle glicêmico, perfil lipídico, creatinina sérica e urinálise, incluindo determinação dos níveis de

albumina urinária. A taxa de filtração glomerular (TFG) foi estimada com base na equação CKD-EPI previamente validada[3]. A proteinúria foi considerada em todos os indivíduos que apresentaram uma relação proteína/creatinina urinária superior a 0,2 mg/g ou uma relação albumina/creatinina superior a 30 mg/g em amostras isoladas de urina[4]. Os grupos de TFGe foram definidos de acordo com o sistema de classificação KDIGO, da seguinte forma: G1 (≥90 mL/min/m^2), G2 (60-90 mL/min/m^2), G3a (45-60 mL/min/m^2), G3b (30-45 mL/min/m^2), G4 (15-30 mL/min/m^2), e G5 (<15 mL/min/m^2). Todas as análises bioquímicas foram realizadas no início do estudo e repetidas conforme apropriado durante o período de acompanhamento.

O desfecho primário foi a diferença entre os grupos sAF na incidência do composto de eventos renais adversos maiores (MAKEs), definidos como o surgimento recente de qualquer um dos seguintes eventos: proteinúria persistente, TFGe < 60 mL/min/m^2, declínio da TFGe de mais de 40% em relação ao valor basal, doença renal em estágio terminal como TFGe < 15 mL/min/m^2 ou início de terapia renal substitutiva e óbito por causa renal[5]. Os desfechos secundários foram: (i) a diferença entre os grupos na alteração média anualizada da TFGe desde o valor basal até a última medição da TFGe; (ii) a diferença na prevalência de declínio rápido, definido como indivíduos com um declínio anualizado da TFGe de mais de 5 mL/min/m^2.[4,6]

Tabela S1. Características basais de acordo com o grupo sAF.

	**sAF**		
	**< 2,85 UA**	**≥ 2,85 UA**	**Valor de p**
n	106	49	
Idade, anos	57 ± 7,3	61 ± 4,9	< 0,001
Sexo masculino, %	63 (57,8)	36 (78,3)	0,015
Duração do DT2, anos	8,54 (10,2)	8,00 (15,1)	0,842
Hipertensão, %	98 (89,9)	43 (93,5)	0,479
Dislipidemia, %	98 (89,9)	40 (87)	0,591

	sAF		
	< 2,85 UA	≥ 2,85 UA	Valor de p
DCV, %	17 (15,6)	13 (28,3)	0,068
Tabagismo, %	4 (3,7)	1 (2,2)	0,630
Ex-fumante, %	44 (40,4)	24 (52,2)	0,176
PAS, mmHg	143 ± 20,9	151 ± 21,1	0,050
PAD, mmHg	85,6 ± 13,3	84,1 ± 10,1	0,631
IMC	30,4 ± 4,61	30,6 ± 4,51	0,805
sAF, UA	2,30 (0,50)	3,25 (0,60)	0,002
Análise bioquímica			
Hb, g/dL	14,3 ± 1,59	14,0 ± 1,39	0,425
A1c, %	8,12 ± 1,64	7,78 ± 1,51	0,227
Colesterol total, mg/dL	178 ± 42,4	178 ± 46,9	0,968
LDL-C, mg/dL	104 ± 35,9	105 ± 37,9	0,879
HDL-C, mg/dL	43,1 ± 10,6	43,2 ± 12,4	0,956
VLDL-C, mg/dL	27 (14,8)	28 (12)	0,628
Triglicérides, mg/dL	169 (131)	171 (88)	0,828
Creatinina, mg/dL	0,90 ± 0,30	0,95 ± 0,21	0,364
TFGe, mL/min			
Basal	87 ± 18,6	83,3 ± 17,3	0,216
Final	88,1 ± 18,4	78,1 ± 19,9	0,004

	sAF		
	< 2,85 UA	≥ 2,85 UA	Valor de p
Alteração	1,08 (1,15)	-5,19 (1,93)	0,019
Classe de TFGe, %			0,616
G1	53 (51,5)	21 (46,7)	
G2	42 (40,8)	21 (46,7)	
G3a	4 (3,9)	3 (6,7)	
G3b	2 (1,9)	0	
G4	2 (1,9)	0	
G5	0	0	
TFGe < 60 mL/min, %	8 (7,8)	3 (6,7)	0,814
Proteinúria, %	21 (20,6)	16 (36,4)	0,071
Classe de albuminúria, %			0,163
A1	44 (40,4)	10 (21,7)	
A2	11 (10,1)	7 (15,2)	
A3	3 (2,8)	2 (4,3)	

DT2: diabetes tipo 2; DCV: doença cardiovascular; PAS: pressão arterial sistólica; PAD: pressão arterial diastólica; IMC: índice de massa corporal; sAF: autofluorescência da pele; UA: unidades arbitrárias; LDL-C: lipoproteína de baixa densidade; HDL-C: lipoproteína de alta densidade; VLDL-C: lipoproteína de muito baixa densidade; TFGe: taxa de filtração glomerular estimada. TFGe e classe de albuminúria de acordo com a classificação KDIGO. DCV para doença coronariana prévia (síndrome coronariana aguda prévia, angina estável ou revascularização do miocárdio) ou acidente vascular cerebral.

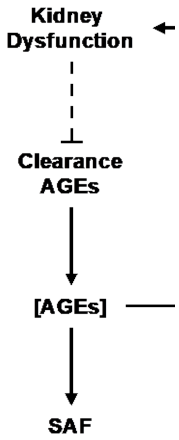

Figura S1. Disfunção renal e sAF.

A disfunção renal atenua o clearance dos AGEs, que perpetuam os desfechos renais e se acumulam na pele, sendo avaliados pela sAF.

REFERÊNCIAS

1. Barreto J, Wolf V, Bonilha I, Luchiari B, Lima M, Oliveira A, et al. Rationale and design of the Brazilian diabetes study: a prospective cohort of type 2 diabetes. Curr Med Res Opin. 2022;38(4):523-9. doi: http://doi.org/10.1080/03007995.2022.2043658. PubMed PMID: 35174749.

2. Fonseca LF, Araujo AB, Quadros K, Carbonara CEM, Dertkigil SSJ, Sposito AC, et al. AGEs accumulation is related to muscle degeneration and vascular calcification in peritoneal dialysis patients. J Bras Nefrol. 2021;43(2):191-9. doi: http://doi.org/10.1590/2175-8239-jbn-2020-0119. PubMed PMID: 33650629.

3. Levey AS, Stevens LA, Schmid CH, Zhang YL, Castro AF 3rd, Feldman HI, et al. A new equation to estimate glomerular filtration rate. Ann Intern Med. 2009;150(9):604-12. doi: http://doi.org/10.7326/0003-4819-150-9-200905050-00006. PubMed PMID: 19414839.

4. Matsushita K, van der Velde M, Astor BC, Woodward M, Levey AS, de Jong PE, et al. Association of estimated glomerular filtration rate and albuminuria with all-cause and cardiovascular mortality in general population cohorts: a collaborative meta-analysis. Lancet. 2010;375(9731):2073-81. doi: http://doi.org/10.1016/S0140-6736(10)60674-5. PubMed PMID: 20483451.

5. Prischl FC, Rossing P, Bakris G, Mayer G, Wanner C. Major adverse renal events (MARE): a proposal to unify renal endpoints. Nephrol Dial Transplant. 2021;36(3):491-7. doi: http://doi.org/10.1093/ndt/gfz212. PubMed PMID: 31711188.

6. Oshima M, Shimizu M, Yamanouchi M, Toyama T, Hara A, Furuichi K, et al. Trajectories of kidney function in diabetes: a clinicopathological update. Nat Rev Nephrol. 2021;17(11):740-50. doi: http://doi.org/10.1038/s41581-021-00462-y. PubMed PMID: 34363037.
