# Supplementary material for: Skin accumulation of advanced glycation end-products predicts kidney outcomes in type 2 diabetes: results from the Brazilian Diabetes Study
Source: J Bras Nefrol. 2024 Aug 23;46(4):e20240047. doi: 10.1590/2175-8239-JBN-2024-0047en (PMC11346773; doi:10.1590/2175-8239-JBN-2024-0047en)
Supplement: Translation

# Translation: Acúmulo de produtos finais de glicação avançada na pele prediz desfechos renais em diabetes tipo 2: resultados do Estudo Brasileiro sobre Diabetes

**DOI:** 10.1590/2175-8239-JBN-2024-0047pt

**Authors:** Joaquim Barreto, Marilia Martins, Cynthia M. Borges, Sofia Helena Vitte, Wilson Nadruz, Rodrigo Bueno de Oliveira, Andrei C. Sposito

**Affiliations:** 1 Universidade Estadual de Campinas, Área de Cardiologia, Laboratório de Aterosclerose e Biologia Vascular, São Paulo, SP, Brasil.; 2 Universidade Estadual de Campinas, Área de Nefrologia, Laboratório para Avaliação do Distúrbio Mineral e Ósseo em Nefrologia, São Paulo, SP, Brasil.; 3 Universidade Estadual de Campinas, Área de Cardiologia, São Paulo, SP, Brasil.

**Keywords:** Insuficiência Renal Crônica, Produtos Finais de Glicação Avançada, Diabetes Mellitus Tipo 2

## Abstract

O acúmulo de produtos finais de glicação avançada (AGEs, do inglês *advanced glycation end-products*) provoca comprometimento morfofuncional dos rins. Os níveis de AGEs podem ser estimados de forma não invasiva por autofluorescência da pele (sAF). Exploramos se a sAF elevada prediz desfechos renais em indivíduos com diabetes tipo 2 (DT2). O estudo foi realizado como uma análise predefinida do Estudo Brasileiro sobre Diabetes, uma coorte prospectiva de centro único de adultos com DT2. Foram considerados os dados de 155 indivíduos acompanhados por até 1.716 dias. A incidência de eventos renais adversos maiores (MAKE, por sua sigla em inglês) foi de 9,6%. Indivíduos com sAF acima da mediana apresentaram maior incidência de MAKEs (4,6% *vs.* 21%; p = 0,002), com HR de 3,39 [IC 95%: 1,06–10,85; p = 0,040] após ajuste por idade e sexo. A alteração média da TFGe ajustada foi de 1,08 unidades (EP: 1,15; IC95%: –1,20, 3,37) no grupo de baixa sAF e de –5,19 unidades [EP: 1,93; IC95%: –9,10, –1,29] no grupo de elevada sAF (diferença entre sujeitos: F: 5,62; p = 0,019). O grupo sAF elevada apresentou maior prevalência de declínio rápido em comparação ao grupo sAF baixa (36,7% *vs.* 15,8%; p = 0,028). Em conclusão, a sAF elevada foi relacionada ao aumento da incidência de MAKEs e ao declínio mais rápido da TFGe entre indivíduos com DT2. Tal fato deve ser considerado pelos profissionais de saúde ao identificar indivíduos mais propensos a complicações renais relacionadas ao diabetes.

## Introdução

A doença renal crônica (DRC) afeta um terço dos indivíduos com diabetes e aumenta o risco de mortalidade e incapacidade^1^. À medida que a prevalência do diabetes aumenta globalmente, ultrapassando atualmente 573 milhões de casos, a identificação de indivíduos predispostos a complicações renais é fundamental para reduzir a mortalidade^2^.

A avaliação dos produtos finais de glicação avançada (AGEs) acumulados na pele, medidos convenientemente por meio da autofluorescência da pele (sAF), surge como uma estratégia promissora para identificar indivíduos diabéticos e não diabéticos em risco de complicações relacionadas aos AGEs^3,4^. Na verdade, dados experimentais anteriores indicam o acúmulo de AGE nos rins, desencadeando apoptose podocitária, comprometimento morfofuncional mesangial e hiperpermeabilidade vascular, todos os quais contribuem para o declínio acelerado da função renal^5^.

No contexto da DRC, o acúmulo de AGE pode ser exacerbado pelo aumento de sua formação devido ao estresse oxidativo e carbonílico, com deterioração concomitante da função renal. Esses AGEs quimicamente estáveis ligados a proteínas de vida longa refletem o estresse metabólico cumulativo, que perpetua o dano celular por meio da modificação direta de proteínas, alterações funcionais ou ligação a receptores específicos de AGEs (RAGEs), induzindo uma série de respostas celulares que culminam em lesões^6^. Consequentemente, os AGEs representam potenciais biomarcadores para a avaliação da progressão da doença renal na DRC^7,8^ (Figura S1).

Apesar disso, o valor preditivo do aumento da sAF para desfechos renais adversos é pouco explorado, particularmente na população brasileira, que apresenta características demográficas, étnicas e epidemiológicas singulares. Para abordar essa lacuna, investigamos a associação entre os níveis de sAF e a incidência de desfechos renais entre indivíduos com DT2 em um centro de pesquisa terciário.

## Métodos

Este estudo foi uma análise predefinida do Estudo Brasileiro sobre Diabetes (clinicaltrials.gov: NCT04949152), uma coorte prospectiva de DT2 realizada por um centro de pesquisa médica no Brasil. O perfil da coorte foi publicado anteriormente e está detalhado no material suplementar^9^. A sAF foi avaliada no início do estudo, enquanto amostras de sangue e urina foram coletadas também no início do estudo e regularmente, a partir de então, para avaliar os desfechos.

O dispositivo AGE-Reader^TM^ (*DiagnOptics BV*, Groningen, Holanda) foi utilizado para estimar a concentração de AGEs na pele com base na fluorescência emitida, que foi calculada em triplicata na face ventral do antebraço^10^. Foram considerados somente os indivíduos com um índice de reflexão cutânea de fototipo de 6% (classe I a IV de Fitzpatrick). Como não existe um valor de referência validado de sAF para nossa população, os participantes foram agrupados como sAF elevada ou baixa com base no valor com maior precisão para o desfecho de interesse na análise da curva ROC (*Receiver Operation Curve*)^10^.

Amostras de sangue total foram coletadas em tubos a vácuo com EDTA de 4 ml e centrifugadas a 3500 rev/min por 10 minutos a 4°C. O plasma foi aliquotado em criotubos e congelado a –80°C até a análise. A taxa de filtração glomerular estimada (TFGe) foi calculada usando os níveis de creatinina sérica e a equação CKD *Epidemiology Collaboration* (CKD-EPI), conforme validado anteriormente^11^. Amostras pontuais de urina foram analisadas para medição dos níveis de creatinina e albumina. A relação albumina/creatinina superior a 30 mg/g foi considerada anormal^12^.

O desfecho primário foi a diferença entre os grupos sAF na incidência de eventos renais adversos maiores (MAKEs), definidos como o surgimento recente de qualquer um dos seguintes: proteinúria persistente, TFGe < 60 mL/min/1,73 m^2^, redução da TFGe de mais de 40% em relação ao valor basal, doença renal em estágio terminal como TFGe < 15 mL/min/1,73 m^2^ ou início de terapia renal substitutiva e óbito por causa renal^13^. Os desfechos secundários foram: (i) a diferença entre os grupos na alteração média anualizada da TFGe desde o início até a última medição da TFGe; (ii) a diferença na prevalência de declínio rápido, definido como indivíduos com uma queda anualizada da TFGe superior a 5 mL/min/1,73 m^212,14^.

### Análise Estatística

As médias foram comparadas pelo teste t de Student quando os dados apresentavam distribuição normal e pelo teste de Mann-Whitney quando os dados apresentavam distribuição não normal. De acordo com o valor de sAF, o *hazard ratio* (HR) de MAKE foi estimado pela regressão de Cox e ajustado por covariáveis. A ANOVA de medidas repetidas foi utilizada para comparar a variação da TFGe entre sujeitos nos grupos de sAF. A alteração anualizada da TFGe entre sua primeira e última medição foi classificada para se ajustar à normalidade e comparada por rANCOVA usando a sAF como variável independente. ROC e a estatística de concordância (C) foram utilizadas para analisar a capacidade da sAF de prever MAKE. Todas as análises foram realizadas usando o SPSS 28.0 versão para MAC, e considerou-se significativo o valor de p < 0,05.

## Resultados

Foram considerados os dados de 155 indivíduos, acompanhados por até 1.716 dias (mediana: 971; IIQ: 550; intervalo: 28–1716 dias). Essa população apresentou média de idade de 58 ± 7 anos, 64% eram do sexo masculino e a duração mediana do DT2 foi de 9 anos (IIQ: 10,6). A prevalência de doença cardiovascular (DCV) prévia foi de 19,4% (18,1% de doença coronariana e 1,3% de acidente vascular cerebral prévio), 3,2% eram fumantes ativos e 91% apresentaram hipertensão. A pressão arterial sistólica e diastólica média foi de 144 ± 21 mmHg e 85 ± 12 mmHg, respectivamente, o índice de massa corporal (IMC) foi de 30 ± 4,6 kg/m^2^, e a hemoglobina glicada (HbA1c) média foi de 8 ± 1,6 %. A TFGe basal foi de 86 mL/min/1,73 m^3^ (grau 1: 50%; grau 2: 42,6%; grau 3A: 4,7%; grau 3B: 1,4%; grau 4: 1,4%), e 14,8% apresentaram albuminúria anormal (A1: 34,8%; A2: 11,6%; A3: 3,2%).

Durante o período de acompanhamento, foram relatados 15 (9,7%) MAKEs: 10 incidentes de DRC (6,5%), 1 (0,1%) declínio da TFGe > 40% e 4 casos de proteinúria de início recente (2,6%).

O grupo MAKE apresentou valores mais elevados de sAF [2,50 (IIQ: 0,7) *vs.* 3,02 (IIQ: 0,8); p = 0,008] e IMC [29 ± 5,6 *vs.* 32 ± 6,8; p = 0,008] e teve maior prevalência de DCV prévia (16,4% *vs.* 40%; p = 0,033) em comparação com o grupo sem MAKE (Tabela 1). Na regressão univariada de Cox, sAF [HR: 2,847; IC 95%: 1,308, 6,197; p = 0,008, por unidade], DCV [HR: 3,23; IC 95%: 1,14– 9,10; p = 0,027] e IMC [HR: 1,142; IC 95%: 1,035– 1,260; p = 0,008] foram correlacionados a MAKE. Além disso, sAF [HR: 3,937; IC 95%: 1,707–9,081; p = 0,001] e IMC [HR: 1,176; IC 95%; 1,064–1,300; p = 0,001], mas não DCV (HR: 2,849; IC 95%: 0,932–8,706; p = 0,066), foram independentemente correlacionados a MAKE após ajuste por covariáveis.

**Tabela 1:** Características basais de acordo com o status do desfecho renal

	Nenhum desfecho renal adverso (n = 140)	Desfechos renais adversos (n = 15)	Valor de p
Idade, anos	59 (11)	63 (10)	0,126
Sexo masculino, %	86 (61)	13 (87)	0,053
Duração do DT2, anos	8,5 (11)	7,6 (11)	0,514
Hipertensão, %	126 (90)	15 (100)	0,199
Dislipidemia, %	123 (88)	15 (100)	0,153
DCV, %	24 (17)	6 (40)	**0,033**
Tabagismo, %	5 (3,6)	0 (0)	0,457
Ex-fumante, %	57 (41)	11 (73)	**0,016**
PAS, mmHg	142 (26)	140 (22)	0,439
PAD, mmHg	84 (17)	86 (10)	0,445
IMC	29 (5,6)	32 (6,8)	**0,008**
sAF, UA	2,5 (0,7)	3 (0,8)	**0,008**
Análise bioquímica			
Hb, g/dL	14 (1,7)	15 (2,5)	0,162
A1c, %	7,6 (1,6)	7,7 (1,7)	0,730
Colesterol total, mg/dL	172 (55)	140 (58)	0,120
LDL - C, mg/dL	100 (41)	75 (50)	0,074
HDL - C, mg/dL	43 (14)	34 (19)	0,852
VLDL - C, mg/dL	27 (12)	32 (11)	0,466
Triglicérides, mg/dL	163 (88)	218 (174)	0,159
Creatinina, mg/dL	0,9 (0,3)	0,9 (0,4)	0,074
TFGe, mL/min	87 (23)	87 (34)	0,187
Classe de TFG, %			0,259
G1	68 (51)	6 (40)	
G2	55 (41)	8 (53)	
G3a	7 (5,3)	0 (0)	
G3b	1 (0,8)	1 (7)	
G4	2 (1,5)	0 (0)	
G5	0	0	
TFGe < 60 mL/min, %	10 (7,5)	1 (7)	0,905
Proteinúria, %	32 (24)	5 (36)	0,348
Classe de albuminúria, %			0,201
A1	52 (37)	2 (13,3)	
A2	15 (11)	3 (20)	
A3	5 (3,6)	0 (0)	

Abreviações: DT2: diabetes tipo 2; DCV: doença cardiovascular; PAS: Pressão arterial sistólica; PAD: pressão arterial diastólica; IMC: índice de massa corporal; sAF: autofluorescência da pele; UA: unidades arbitrárias; LDL - C: lipoproteína de baixa densidade; HDL - C: lipoproteína de alta densidade; VLDL - C: lipoproteína de muito baixa densidade; TFGe: taxa de filtração glomerular estimada. Notas: TFGe e classe de albuminúria de acordo com a classificação KDIGO^7^. DCV para doença coronariana prévia (síndrome coronariana aguda prévia, angina estável ou revascularização do miocárdio) ou acidente vascular cerebral.

A sAF apresentou uma área sob a curva (AUC) de 0,786 (EP: 0,067; IC 95%: 0,654–0,917; p < 0,001) para MAKE. A estatística K-S retornou o valor de corte ideal de sAF de 2,85 UA (sensibilidade: 80%, especificidade: 26%) e um teste não paramétrico máximo de Kolmogorov-Smirnoff de 0,538, indicando que a sAF foi melhor em prever desfechos renais para essa população do que um modelo aleatório.

Com base nesse achado, agrupamos os pacientes em sAF elevada (n = 46; ≥2,85 UA) e sAF baixa (n = 109; <2,85 UA). Em comparação com o grupo sAF baixa, o grupo sAF elevada era mais velho (57 ± 7 anos *vs.* 61 ± 4,9 anos; p < 0,001) e apresentava maior frequência de homens [57,8% *vs.* 78,3%; p = 0,015] (ver Tabela S1). O grupo sAF baixa apresentou 5 (4,6%) e o sAF elevada apresentou 10 (21,7%) MAKEs (p = 0,002), com HR de 4,58 (IC 95%: 1,56– 13,46; p = 0,006) no modelo não ajustado e HR de 3,39 [IC 95%: 1,06 – 10,85; p = 0,040] após ajuste por idade e sexo (Figura 1). Ajustes adicionais por pressão arterial sistólica e HbA1c, que são fatores de risco conhecidos para MAKEs, não anularam a influência da sAF elevada nos desfechos [HR: 5,28; IC 95%: 1,44, 19,37; p = 0,012].

**Figura 1:** Sobrevida livre de eventos renais conforme grupo de sAF.

No início do estudo, a TFGe foi comparável entre os grupos sAF. Os níveis de TFGe permaneceram inalterados no grupo de baixa sAF (87 + 18 unidades *vs.* 88 + 18 unidades; p = 0,078), enquanto a TFGe diminuiu significativamente entre os indivíduos com sAF elevada (83 + 17 unidades *vs.* 78 + 19 unidades; p = 0,025). A alteração média da TFGe ajustada foi de 1,08 unidades (EP: 1,15; IC de 95%: –1,20, 3,37) no grupo sAF baixa e de –5,19 unidades [EP: 1,93; IC de 95%: –9,10, –1,29] no grupo sAF elevada (diferença entre sujeitos: F: 5,62, p = 0,019) (Figura 2). O grupo sAF elevada apresentou maior prevalência de declínio rápido em comparação ao grupo sAF baixa (36,7% *vs.* 15,8%; p = 0,028).

**Figura 2:** Alteração média ajustada na taxa de filtração glomerular de acordo com os grupos sAF.

## Discussão

O acúmulo de AGEs na pele oferece um método viável e reproduzível para identificar indivíduos com maior risco de desfechos renais adversos. Nosso estudo demonstrou que valores elevados de sAF foram um fator de risco independente para MAKEs e predisseram um declínio mais rápido da TFGe. Esses achados se alinham com relatos anteriores de estudos retrospectivos realizados em países orientais, que mostraram que a taxa de declínio da TFGe e o incidente de doença renal em estágio terminal estão associados a valores mais elevados de sAF tanto em indivíduos sem DT2 quanto em indivíduos com DT2 com DCV estabelecida^8,15^. Como a progressão do comprometimento renal varia significativamente entre os países e é influenciada pela prevalência de DCV, nossos dados complementam resultados anteriores, uma vez que validam essa relação em uma população etnicamente diversa com um espectro mais amplo de risco cardiovascular^16^. Os valores de sAF podem variar significativamente entre as populações, o que ressalta a necessidade de valores de referência específicos para cada região, como demonstrado por pesquisas anteriores^17^.

Em estudos anteriores, o valor preditivo da sAF foi descrito para complicações do diabetes. Boersma et al.^18^ confirmaram que a sAF prediz DCV e mortalidade em um grupo maior de 2.349 pessoas com DT2. A sAF também mostrou uma associação mais forte com eventos cardiovasculares (ECV) futuros e mortalidade do que os níveis de colesterol ou pressão arterial. A insuficiência renal é outra condição comum associada ao acúmulo de AGEs e está clinicamente associada a um forte aumento na taxa de complicações cardiovasculares. A sAF também foi identificada como um preditor independente e robusto de mortalidade. Em comparação com as variáveis do mecanismo de risco do *UK Prospective Diabetes Study* (UKPDS), a sAF foi considerada o preditor individual mais eficaz de mortalidade total e cardiovascular após a idade, adicionando valor preditivo ao mecanismo de risco do UKPDS, resultando em reclassificação de risco em 25–30% dos pacientes^19^.

Estudos iniciais sobre a sAF na insuficiência renal relataram níveis aumentados de sAF em pacientes com insuficiência renal moderada até pacientes em hemodiálise e confirmaram a sAF como um preditor de risco forte e independente para mortalidade em pacientes com insuficiência renal^20,21^ e em pacientes transplantados^22^. Arsov et al.^23^ em 2014 e Crowley et al.^24^ em 2013 encontraram níveis de sAF fortemente aumentados em pacientes em HD. Nesse último estudo, os níveis de sAF foram 64% mais elevados do que nos controles pareados por idade, com uma redução nos níveis de sAF após o transplante renal. Em resumo, indivíduos com DT2 com valores mais altos de sAF correm maior risco de desenvolver MAKE. São necessários mais estudos para determinar se o tratamento para reduzir o acúmulo de AGE resulta em melhores desfechos renais.

Nosso estudo teve limitações. Primeiramente, o tamanho da amostra foi pequeno em comparação com estudos anteriores, resultando em um número limitado de eventos, o que pode ter afetado o poder estatístico. Em segundo lugar, o valor de corte utilizado para a sAF pode não ser aplicável a outras populações, uma vez que essa variável é suscetível à influência de características demográficas que variam significativamente entre os estudos. Por fim, embora a taxa de declínio anualizada da TFGe seja mais precisa quando os valores da TFGe são medidos em intervalos regulares, calculamos sua inclinação com base nas medidas de creatinina sérica avaliadas oportunisticamente durante o estudo.

## Material Suplementar

O seguinte material online está disponível para o presente artigo:

Métodos Suplementares - Estudo Brasileiro sobre Diabetes.
